# Study on the integrated teaching of English education, mental health and students' wellbeing

**DOI:** 10.3389/fpsyt.2022.953325

**Published:** 2022-07-25

**Authors:** Qian Zhao, Hang Shang

**Affiliations:** ^1^Faculty of Teacher Education, Pingdingshan University, Pingdingshan, China; ^2^Basic Teaching Department, Liaoning Technical University, Fuxin, China

**Keywords:** English intentions, student behavior, PLS-SEM, wellbeing, student orientation, mental health, teacher behavior

## Abstract

Teachers' attitudes, behavior, and practices play an integral role in enhancing the students' learning activities. Teachers' strategies ensure the individuals' professional development by creating a healthy learning environment. The study's primary objective is to analyze students' attitudes toward learning English as a foreign language. The data was collected from the 359 colleges and universities students by adopting a convenient sampling technique. The study shows English self-concept and teacher behavior student orientation significantly positively impact attitudes toward learning English as a foreign language. Motivation for English and English Intentions mediate the relationship between English Self-concept, teacher behavior-student orientation, and Attitude toward learning English as a Foreign Language. The study motivates future studies to focus on the EFL, individuals' learning motivation and intentions in other domains across diverse contexts.

## Introduction

In the globalized world of multilingual societies, the global education system has caused English to extend its spectrum from a common language to international reach. Today, English, holding a prominent linguistic position, has significantly become a vital spread serving worldwide communities. Its growing popularity has made it an imperial tool to foster global learning activities, substantially becoming a paramount requirement of today's academia (1). In particular, this language of academics has profoundly touched the cross-cultural boundaries with its foreign significance.

In education, the students face considerable challenges in adjusting to the increasing demand of the ill-structured learning environment. These emerging difficulties affect the students' mental health and wellbeing. Given the articulation, the study shows that interest in students' mental health and wellbeing has grown exponentially in recent years (2). A healthy mind enhances the students' learning activity, thus ensuring their wellbeing. This widespread agreement (i.e., English) in education offers a unique opportunity to promote students' mental health and wellbeing, thus encompassing the academic professionals to create a healthy learning environment for language learning. However, the absence of this concept may lead the students to face difficulties and challenges, thus hampering the students' English learning process and academic succession (3). In this regard, the institutions have acknowledged students' emotional and psychological wellbeing to be essential in improving individuals' learning process.

Indeed, this matter of students' health and wellbeing holds a profound prominence in fostering the students' learning. Undoubtedly, today, English has gained massive foreign popularity. Its growth in education has made it function as an international tool for fostering learning outcomes (4). Learning English as a foreign language has become the imperial requirement of today's learners. In recent years, English language evolution has shifted the students' focus toward its adaption. Therefore, understanding students' attitudes as an influential factor have become essential in strengthening language learning pedagogy. Students' attitudes form an integral factor influencing English language learning. Given the explanation, the study shows that English foreign efficiency has encouraged students to learn it as an essential requirement for academic achievement (5).

In language acquisition, an individual's success largely depends on their self-concept. Self-concept is a dynamic system of an individual's learned beliefs and actions (6). The self-concept positively enhances the learning process, substantially making students evaluate their capabilities and achievements (7). It facilitates the students' learning, thereby identifying the areas for improvement. It promotes self-regulating activities evaluating individuals' skills and competencies. Accordingly, concerning EFL, the study shows that a positive self-concept provides information on individuals' strengths and weaknesses (8), thus enabling them to assess their need for a foreign language (e.g., English).

Also, the study shows that the teachers' positive behavior significantly affects the students' academic success, learning intention, and achievement (9). Teachers' attitudes, behavior, and practices enhance students' learning activities. Teachers' strategies ensure the individuals' professional development by creating a healthy learning environment. The study shows that the teachers' behavior boosts the students' orientation (i.e., motivation and achievement) (10). Teachers' pedagogical characteristics are the most influential factors motivating language learning. Effective English learning enables the students to direct their learning outcomes and motivation (11).

Significantly, besides teachers' having responsibility for influencing the students learning activity, student learning motivation also works as a critical factor in ensuring successful learning. Motivation is a primary source affecting the students learning. Given the illustration, the research shows that the lack of motivation makes the students quit learning English (12). Perhaps, motivation infuses the success of the foreign language. Students enjoy learning when they feel motivated regarding foreign languages. Accordingly, the study reveals that with the increasing role of English as a foreign language, motivation has become a key driver in improving students' learning (13).

Indeed, today, the power to master the English language has gained considerable academic attention (14). Presently, non-English individuals have found striving hard to improve their education in the English language. Learning English as a foreign language is crucial for advancing today's learning environment. Unsurprisingly, today, English has made the majority of non-speakers interested in learning EFL. Its progressing international value has led the world's population to realize its benefits, subsequently holding positive intentions toward language acquisition. Given the articulation, the study shows that English rapid acceleration has demanded learners of different nationalities to shift their focus to English learning (15), thereby achieving positive learning outcomes.

Given the evidence, the literature showed that a high motivation boosts the students' intention to learn new concepts. However, besides this favorable relation, the study revealed that motivation to learn English as a foreign language has constantly faced backlash from the students (16). Indeed, English as a foreign language eases a gap in student motivation and their intention to learn the language. Hence, a lack of consensus on the prestigious usage of the potential English language has still massively recorded. The research states that besides the increasing significance of the English language, most schools are not using English as the home language (17). Perhaps, the research on foreign language learning had caught little academic attention. Therefore, to fill the research gap, this study recommends accepting English as a foreign learning requirement for today's learners.

However, against this drawback, the main objective of the present study is to investigate the factors stimulating the students' attitude toward learning English as a foreign language. The study expects that studying these factors will lead the students to embrace English learning as an international language fostering self-concept and teachers' behavior student orientation. Moreover, the study also highlights the mediating effect of motivation and English intention against EFL (i.e., attitude toward learning English).

Therefore, this incorporation appears to be a potential benefit in advancing the education sector. These insights develop a valuable contribution to the world's education sectors (i.e., eastern and western). In particular, the existing research gap showed that interest in the factors such as motivation and intention call for further investigation in the context of learning the language. Significantly, the study holds immense importance by empirically exploring the trajectories influencing students' perception of EFL learning. However, irrespective of specific influence, the unique research incorporated combinations of factors, thus elevating positive learning outcomes.

Significantly, this knowledge of English proficiency provides distinctive characteristics to students by making them essentially advance their careers through effective English learning. The study offers the learners a comparative opportunity to experience EFL learning. It suggests improving the English learning motivation, self-concept, and intention to learn English to gain effective learning outcomes. Furthermore, the research findings also allow the educationists and government to understand the benefit of learning the English language, thus optimally supporting the English teachings. In particular, the research findings suggest adopting high linguistics teaching skills, thereby improving motivation and attitude.

As a quick reminder: this current paper has split into six different sections where the proceeding section highlights the research background. Similarly, Section Methodology elaborates on the research methodology, and section Results and discussion documents the research outcomes. Lastly, section Conclusion discusses the study outcomes, with section Conclusion concluding the research paper.

## Literature review

### English self-concept and attitude toward EFL

English, a global language, enhances the learners' fluency by significantly advancing their learning activity (18). Today, English, a profound widespread, had direct individuals' attitudes toward its learning. In education, attitude is a fundamental component regulating students' learning. Perhaps, attitude plays a significant role in promoting English as a foreign language (EFL). A favorable learning attitude leads to a positive learning orientation. Based on this statement, the study shows that attitude plays a primordial role in fostering the individual's English learning (19).

Individuals' success largely depends on the mastery of the English language, where positive self-concept plays an integral role in influencing the individual's learning attitude. The students' learning perception (i.e., self-concept) helps them assess their need for EFL. In support, the study shows that the language self-concept provides feedback to the students, fundamentally shifting their focus toward EFL (20). The positive English self-concept enables the learners to display a favorable work orientation. The English self-concept makes the learners assess their foreign language skills by identifying the areas for improvement. This profound notion of evaluating perceived strengths and weaknesses in English makes the young learner embrace the self-concept as a dominant tool in promoting foreign language education (8). In particular, self-concept assessments shift the learners' focus toward English. Given the illustration, the study shows that English self-rated knowledge and skills provide students with a positive learning experience, thus directing their attention toward the English language (16). Moreover, in exploring the attitude of Palestine school graduates, the study reveals that students have shown the highest attitude toward English by accepting it as a foreign learning language (21). Therefore, based on the above considerations, we have devised the following hypothesis.

*H1: English Self-concept has a positive and significant impact on Attitude toward learning English as a Foreign Language*.

### Teacher behavior-student orientation and attitude toward EFL

With the changing paradigm of global education, exploring teacher behavior has become an independent area demanding the establishment of English language teachings. The EFL has highlighted the teachers' role in advancing foreign language teachings from local to international settings. In support, the study from Turkey showed that teachers adopting novel teaching programs had significantly addressed the students' EFL learning needs (22). Perhaps, acquiring the English skills demands the students to gain fluency in the language, expressing this phenomenon as a fundamental tool driving the learning process. This teachers' support enables the learners to accept English as an essential driver influencing academic success. Accordingly, the study reveals that the Malaysian education system has encouraged teachers to introduce innovative teaching strategies to advance students' English learning (23).

Students' learning depends on the teachers' teaching methods, style, and practices. Significantly, the instructors teaching material act as a guide in assisting the students learning activities. In this regard, today, teacher behavior- student orientation is gaining growing attention in shaping the students' attitude toward their studies. Teachers' teaching strategies fundamentally work to improve the students learning process. Accordingly, in support, the study shows that teachers' English learning strategies have improved the proficiency of the technical learners, thus directing their focus toward English learning (24).

Altogether, teachers have a responsibility to influence student learning orientation. The teacher's positive emotion toward EFL elevates the need for an effective curriculum to strengthen the students' skillset. Teachers' constructive feedback and assignments improve students' understanding of English subjects, thus gaining academic success. The study shows that teachers providing the necessary learning materials develop an EFL environment (e.g., books, lecturers, feedback) (25), substantially assisting the students' academic progress. However, this fundamental teacher-student orientation approach elevates the EFL among the students. In explaining this notion, the study shows that teachers' classroom materials improve the students' understanding of English vocabulary, thus making it a profound learning medium fostering student attitude and engagement (26). Consequently, the prior studies conclude.

H2: Teacher Behavior Student Orientation has a positive and significant impact on Attitude toward learning English as a Foreign Language.

### English self-concept and motivation for English

In the rapidly evolving learning climate, a positive self-concept boosts the students' confidence in the work activity. The self-concept motivates individuals to achieve good academic grades at all educational levels. English is the most important foreign language that has become the core for accelerating learning activities. Learning English requires getting knowledge about the subject. However, today, the motivation to learn English as a foreign language demands global attention. The self-concept makes the students identify the reason behind studying the English language, ultimately boosting their learning motivation (27).

Indeed, academic self-concept predicts the students' motivation for learning. Given the illustration, the study shows that Self-concept increases individuals' autonomy, competence, and motivation (28). Undoubtedly, this multidimensional concept influences individual learning ability and academic choices. The self-concept assessment makes individuals participate in the learning activity. Perhaps, it is an effective tool for ensuring the student's active involvement. Given the articulation, the study states that in improving language skills, self-concept learning motivates the students to accomplish learning goals (29). Altogether, English is a generic language that has gained massive popularity over the years. Perhaps, the literature reveals that in recent years, students have boosted their morale in learning this prestigious historical configuration. Accordingly, the study shows that a positive self-concept makes the student enjoy the course, thus enhancing their motivation in the subject (30). Subsequently, the prior research findings made us conclude the following hypothesis.

H3: English Self-concept positively and significantly impacts motivation for English.

### Teacher behavior- student orientation and English intentions

The EFL demands the teachers' attention, involvement, and guidance in executing the learning activity. The teacher-behavioral-student orientation regulates the individuals' goal (i.e., learning the foreign knowledge skill), fostering their desire to gain language proficiency (31). In the learning process, students holding different perspectives exhibits diverse behavioral responses (e.g., motivation, intention, orientation, emotions). Accordingly, EFL anxiety has extensively gained researchers' attention. The foreign language learning anxiety discourages students' desire to learn the new language. However, in such situations, the teachers' support lowers the students' learning anxiety by making them effectively involve in the EFL process. In the illustration, the study performed on the Chinese undergraduate student showed that EFL is the product of teacher guiding behavior, while language learning anxiety is highly related to learners' perception of the subject (32).

Undoubtedly, today, instructors widely contribute to students' learning process. The teachers provided curriculum helps the students to understand the challenging concept. English is a complex language that demands teachers' assistance. Teachers' behavior-student orientation allows teachers to make learning simple and easy with their teaching skills. Based on this statement, the research shows that teachers' teaching boosts students learning intention, thereby fostering the process of learning a language (33). Verily, the English learning intention is the most needed phenomenon guiding the EFL process. Teachers' behavior plays an essential role in boosting the students' desire to learn English as a foreign language. In the explanation, the study revealed that by creating a positive learning environment in China, the students were most likely to enjoy EFL learning through the support of their teachers, substantially raising their interest and passion for EFL teaching (34). Consequently, the prior literature findings propose.

H4: Teacher Behavior Student Orientation positively and significantly impacts English Intentions.

### English self-concept and English intentions

Most students learn English because they believe it plays a paramount role in advancing their educations careers. However, without understanding the language's significance, students lose hope in learning the language. Undoubtedly, the English language has received global popularity by significantly making multinational students realize its benefit. A positive self-concept is a critical component of students' achievements and success. Given the illustration, the study shows that by recognizing the value of a foreign language, the students of Arab countries have significantly shifted their attitude toward learning English as a foreign language (35).

The academic self-concept influences one's interest in the subject. In recent years, this idea fundamentally works well in education, especially in English. English self-concept makes students learn new vocabulary, thus performing well in every sphere of life. Given the illustration, the study from Vietnam showed that students' perception influences their career interest in EFL, thereby increasing their career intention in English (36). Consistently, the study shows that self-concept natures the students' academic careers and their intention to pursue the course in the future (37). Self-concept is a motivational component that enhances students' language skills and academic choices. Accordingly, the study performed on Indonesian students revealed that self-belief is a motivational factor fostering the individual's aptitude to learn English as a foreign language (38). Subsequently, the hypothesis developed from the previous literature suggests.

*H5: English Self-concept has a positive and significant impact on English Intentions*.

### Teacher behavior-student orientation and motivation for English

Motivation as the instrumental factor acknowledges the need for acquiring English as a foreign language. Teachers' behavior- student orientation, an integrative approach, makes the students study English with more motivation and enthusiasm, thus influencing their academic choices. Motivation to learn English as an international language is highly related to teaching aspects (e.g., behavior, curriculum, activities) (39). Today's language motivation and teachers' behavioral orientation are necessary to achieving effective academic outcomes. Students generally learn English to obtain their purpose. Teachers' supporting students' goals enhances their motivation, thus ensuring their learning progression (40). Indeed, this makes learning motivation essential to achieving educational success. Boredom is an unpleasant feeling associated with individual learning. This psychological emotion makes the student disengage from the learning process, thus lowering their EFL motivation (41). However, this study calls the teachers to support the students learning by motivating them to indulge in the English learning process. Perhaps, teachers guiding the students' work orientation require effective teaching strategies, thus facilitating their learning motivation. Given the explanation, the study shows that learners' motivation and teacher behavior encourage non-Latin individuals' (e.g., Korean, Japanese) to learn English as a foreign language (42). Hence, based on the previous literature, the hypothesis proposes.

*H6: Teacher Behavior Student Orientation positively and significantly impacts Motivation for English*.

### The mediating role of motivation

Motivation develops a strong bond with students' learning attitudes. Academic progress highly depends on students learning motivation and their interest in language. The students come to the classroom to learn something new. However, understanding these motives increases their morale by realizing the worth of studying the language. Generally speaking, motivation plays an integral role in advancing English learning as a foreign language. English boosts the students' motivation and learning attitude. Given the explanation, the study indicates a positive relationship between student motivation, learning attitude, and EFL (43).

The acquisition of English supports both the teaching and learning orientation. Perhaps, learning this language demands individuals' time, attitude, and motivation. Aptitude and motivation are effective drivers of developing language skills (44). Indeed, to learn the English language, individuals should develop a positive attitude regarding its increasing acceptance. Positive learning motivation enables individuals to gain mastery over a foreign language. In this process, the self-concept helps individuals evaluate their language abilities, thus gaining enhanced language proficiency. The self-concept provides autonomy to individuals' making them self-evaluate their competency level. Given the illustration, the research shows that self-concept strengthens the learners' skills and motivation, thus developing English as an international language (45).

In the same vein, motivation leads the individual to gain language proficiency. It helps the students to raise their competency to an expert level. Accordingly, the study shows that the significant shift in language learning has made the teachers' characteristics increase the students' participation (46), thus enabling them to obtain expertise in all aspects (i.e., reading, writing, communicating). Teachers play an integral role in fostering the students learning with their unique teaching strategies. The study performed on the Chinese EFL students showed that teachers' foreign learning programs impact the students learning attitudes (47). It enhances students' language development skills, thus promoting the teachers' language guidance (e.g., instructions, practices) to nurture classroom learning (48). Likewise, the study determining the effect of teachers' behavior on student attitude states that teacher behavior reports students' active participation in class classroom (49). Furthermore, the study also shows that the immediate feedback provided by the teachers encourages and motivates the individual to learn English through the support of teachers' provided materials (50). Hence, based on the above literature, the hypotheses suggested are as follows:

*H7: Motivation for English has a positive and significant impact on Attitude toward learning English as a Foreign Language*.

*H7(a): Motivation for English mediate the relationship between Self-concept of English and Attitude toward the learning of English as a Foreign Language*.

*H7(b): Teacher Behavior- Student Orientation mediates the relationship between English Self-concept and Attitude toward the learning of English as a Foreign Language*.

### The mediating role of English intention

Fundamentally, English as a medium of language has grown over time, thus fostering the students' professional activities. The EFL inspires the students to learn the language at all school levels. It is an instrumental motivator encouraging the students to involve in EFL learning. Given the explanation, the study shows that EFL positively influences the students' attitudes toward English (51). The study reveals that EFL inspires learners to enjoy English teaching by realizing its value and benefit (52). Given the illustration, the research on Chinese EFL students showed that students learning enjoyment were highly related to the students' motivation, interest, confidence, and aptitude (53).

Moreover, the study also shows that EFL motivates the students to obtain positive learning outcomes by strongly shifting their focus toward English (54). The self-concept improves the learners' skills and understandings, thus increasing their intention to learn a new language. Given the explanation, the study shows that EFL learning increases student motivation, thus their learning intention (55). Similarly, the study revealed that Japanese female students showed a high EFL intention (56) during their language learning class. Indeed, English has bloomed as a vital need for today's education system. But besides this, students are fundamentally facing complications and difficulties in their learning. Based on this notion, the study on 145 Indian students revealed a direct correlation between student self-concept (i.e., belief, perception) and student intention of EFL (57, 58).

Additionally, by understanding the language learning belief, teachers provide instrumental support to their students' by arranging sufficient teaching materials for their assistance (e.g., curriculum, syllabus, policies). However, the strength of the educational system cannot be judged without the EFL. Therefore, in coping with the growing educational requirement, the study showed that innovative teaching methods have rigorously improved the students' English reading and writing skills, thus making them confident in the learning methods (59, 60). Hence, these teaching methods and strategies foster students learning experiences to increase their motivation and interest in the language. Hence, it is high time to realize the value of learning English as a foreign language. In the fast-growing educational environment, the new spread aids the students learning process. Accordingly, teachers have adopted novel teaching methods (e.g., technology) to improve the students' language learning skills. Given the articulation, the study showed that these teaching behaviors enhance students' intention to learn English as a foreign language (61).

*H8: English Intentions have a positive and significant impact on Attitude toward learning English as a foreign language*.

*H8(a): English Intentions mediate the relationship between English self-concept and attitude toward the learning of English as a foreign language*.

*H8(b): English Intentions mediate the relationship between teacher behavioral student orientation and attitude toward the learning of English as a foreign language*.

**Figure 1:**
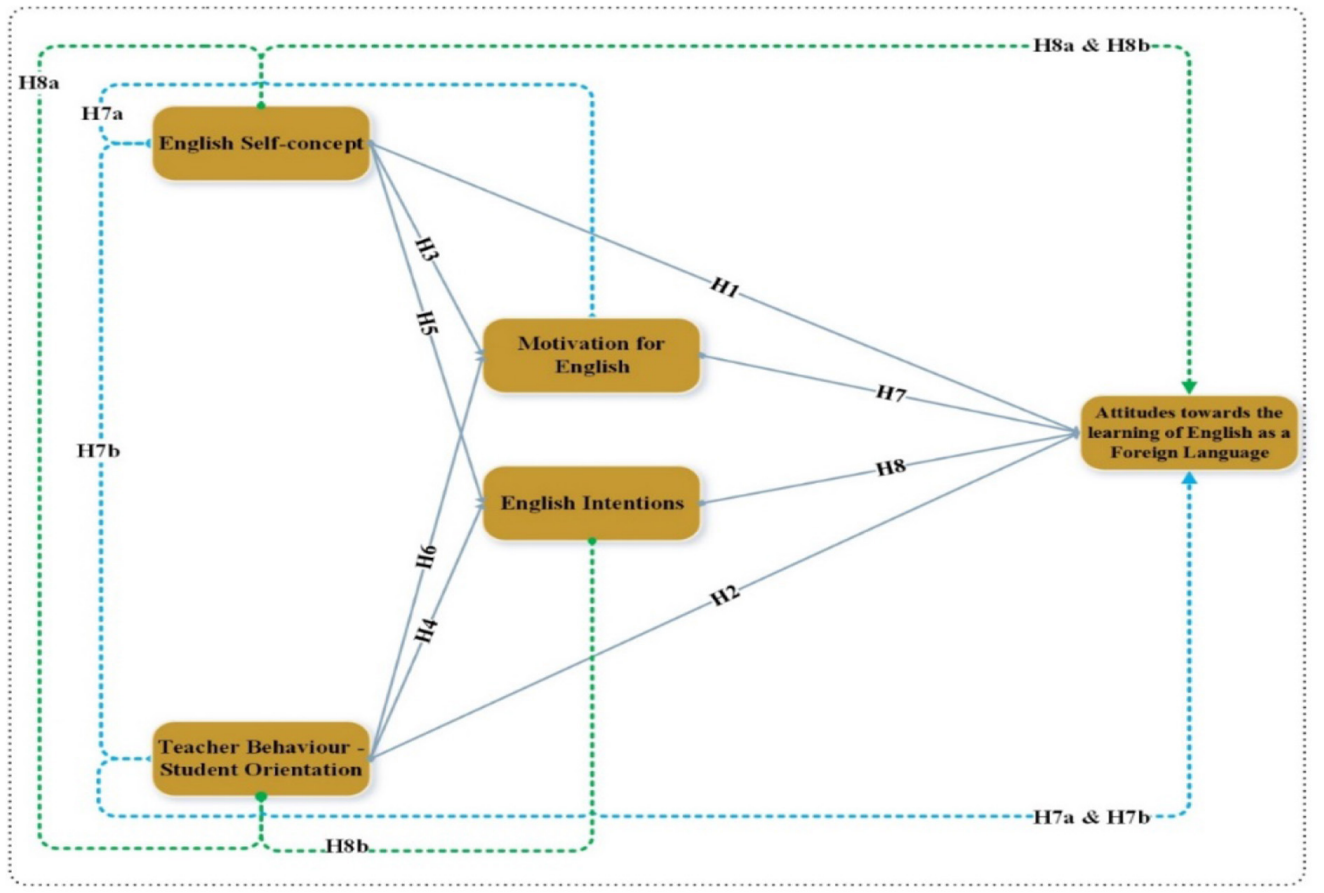
Conceptual framework.

Figure 1 shows the study hypothesis relationship, which includes independent, dependent and mediating variables.

## Methodology

The study used quantitative research to examine the impact of English self-concept, and Teacher behavior student orientation on the attitudes toward learning English as a foreign language. Additionally, the study examines the mediating effect of motivation for English and English intentions between English self-concept, teacher behavior and attitudes toward learning English as a foreign language. The study utilized the positivism philosophy, which means data gained by scientific observations and measurements. The deductive reasoning approach is applied in this study. The data was collected from the students studying in the Colleges and Universities of China. The study adopted the convenient sampling technique for the data collection process. Previously developed structured questionnaires were used for data collection.

Moreover, the five Likert scales were used to measure the survey, ensuring the reliability and validity of items. However, before sending the questionnaires to respondents, a cover letter is attached to ensure the confidentiality of respondents for research objectives. The sample of 430 questionnaires was distributed to the students at different colleges and universities in China, and data was collected electronically. After that, 400 questionnaires were received, and finally, 359 questionnaires were chosen to analyze the data with an 89% response rate. The study used the smart PLS for the Partial least square's structural equation model.

Table 1 illustrates that the study data collected data from the higher educational institutes where numerous respondents actively participated in the research program. A massive positive response has gathered during the survey. During the data collection, the students were asked different questions, thus providing them with the opportunity to understand and analyze every bit of the research topic. The candidates increased our response rate by sincerely answering the questions in a non-biased way. Altogether, out of 400 questionnaires, 359 candidates were selected based on our criteria. Among them, 179 were males, and 180 were females. Upon identification, all the participants belonged to different age groups ranging from 18 to 30 years. Moreover, the study collected data from diverse groups of classes (i.e., intermediate, bachelor, master, and M- Fill). Indeed, this fundamental demographic made us successful in achieving organic data from a large population.

**Table 1:** Demographic characteristics.

**Items**	**Frequency (*****N*** = **359)**	**(%)**
**Gender**		
Male	179	49.9
Female	180	50.1
**Age**		
18–20	51	14.2
21–23	130	36.2
24–26	129	35.9
27–30	49	13.6
**Class**		
Intermediate	27	7.5
Bachelor	154	42.9
Master	146	40.7
MPhil/Others	32	8.9

### Common method bias

Common method bias using Harman's single-factor approach was applied in this study. The variance extracted by one single factor is 11.961% which is <50%, indicating no common method bias (62).

## Results and discussion

### Assessment of measurement model

Reliability, validity, and discriminant validity were analyzed in the measurement model. In reliability, the Alpha and CR values must be investigated, and both must be higher than 0.6 (63, 64). Convergent validity includes the standardized loadings of each construct analyzed, which are also higher than 0.5 (65). Furthermore, the Average Variance Extracted (AVE) is also found to be >0.5, resulting in no convergent validity issue in this research (see Table 2). English Self-Concept alpha value was 0.861.

**Table 2:** Reliability and validity analysis.

**Construct**	**Items**	**Loading**	α	**CR**	**AVE**
English self-concept	SCE_1	0.803	0.861	0.861	0.554
	SCE_2	0.722			
	SCE_3	0.744			
	SCE_4	0.745			
	SCE_5	0.703			
Teacher behavior student orientation	TBSO_1	0.746	0.835	0.835	0.559
	TBSO_2	0.746			
	TBSO_3	0.738			
	TBSO_4	0.759			
Motivation for English	ME_1	0.685	0.824	0.824	0.540
	ME_2	0.736			
	ME_3	0.731			
	ME_4	0.785			
English intentions	EI_1	0.759	0.867	0.866	0.566
	EI_2	0.802			
	EI_3	0.760			
	EI_4	0.766			
	EI_5	0.666			
Learning English as a foreign language	SALEFL_1	0.788	0.922	0.922	0.543
	SALEFL_10	0.768			
	SALEFL_2	0.707			
	SALEFL_3	0.778			
	SALEFL_4	0.712			
	SALEFL_5	0.754			
	SALEFL_6	0.703			
	SALEFL_7	0.741			
	SALEFL_8	0.687			
	SALEFL_9	0.721			

As shown in Table 3, the square root of AVE must be greater than the correlation of the coefficient, which indicates a good discriminant (66). The second cross-loading method was used to evaluate the discriminant. The outcomes illustrate that there are no cross-loadings found between the items.

**Table 3:** Discriminant validity analysis (Fornel Larcker and HTMT).

**Constructs**	**1**	**2**	**3**	**4**	**5**
1. English Intentions	0.752	0.627	0.701	0.623	0.598
2. Motivation for English	0.629	0.735	0.707	0.643	0.617
3. Learning English as a Foreign Language	0.704	0.709	0.737	0.693	0.670
4. English Self-Concept	0.626	0.645	0.695	0.744	0.640
5. Teacher Behavior Student Orientation	0.598	0.617	0.670	0.640	0.747

*Values on the diagonal (bold) represent the square root of the average variance extracted, while the off diagonals are correlations*.

This research also checks the VIF values for English intentions, motivation for English, and learning English as a foreign language. English self-concept and teacher behavior student orientation. All values fall below the threshold (see Table 4).

**Table 4:** Variance influence factor.

**Constructs**	**1**	**2**	**3**	**4**	**5**
1. English intentions			2.022		
2. Motivation for English			2.137		
3. Learning English as a foreign language					
4. English self-concept	1.694	1.694	2.204		
5. Teacher behavior student orientation	1.694	1.694	2.034		

**Figure 2:**
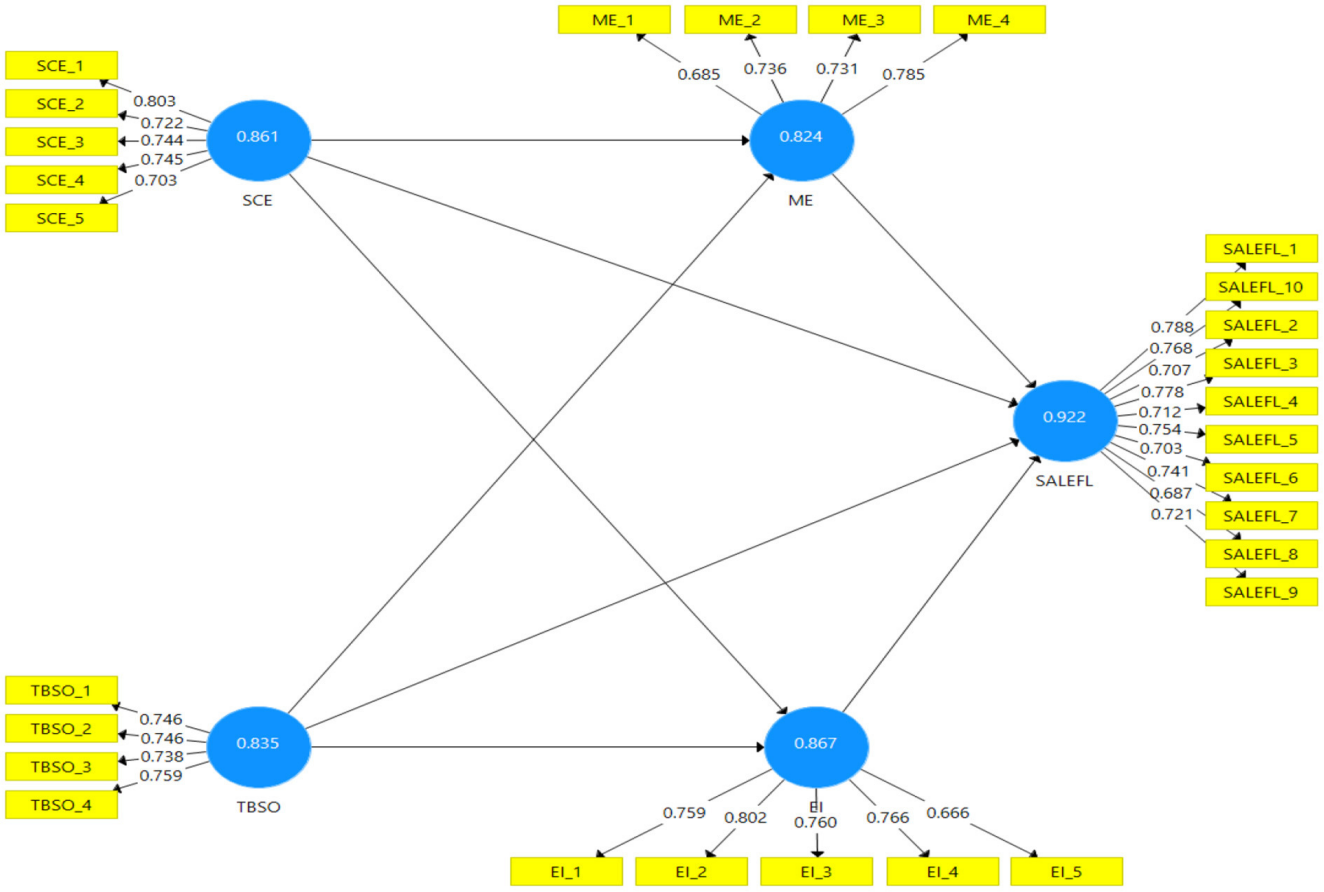
Assessment of measurement model.

Figure 2 presents the graphical representation of the assessment measurement model. All the variable's factor loadings values are higher than 0.6. The item EI_5 has the lowest factor loading value of 0.666, while the item SCE_1 has the highest factor loading value of 0.803.

### Structural model

This research applied the PLS-SEM technique using Smart-PLS software version 3.3.3 for hypothesis testing (67). The bootstrapped technique was used by recommending a 5,000-sample size to acquire the hypothesis testing results. Table 5 provides the results of the direct hypothesis relationship between study variables.

**Table 5:** Hypotheses testing direct effect.

**Hypothesis**	**Direct**	**Std**.	**Std**.	**T**	**P**
	**Relationships**	***Beta***	**Error**	**Values**	**Values**
H1	SCE → SALEFL	0.222	0.059	3.775	***
H2	TBSO → SALEFL	0.194	0.075	2.583	*
H3	SCE → ME	0.423	0.078	5.393	***
H4	TBSO → EI	0.335	0.082	4.09	***
H5	SCE → EI	0.411	0.082	5.021	***
H6	TBSO → ME	0.347	0.084	4.104	***
H7	ME → SALEFL	0.271	0.096	2.808	**
H8	EI → SALEFL	0.279	0.087	3.214	**

^*^Indicates significant paths: *p < 0.05, **p < 0.01, ***p < 0.00.

On Attitude toward learning English as a Foreign Language was accepted and confirmed with a standardized path coefficient of 0.222. The impact of English Self-concept on English Self-concept is positively significant at the 0.001 level. H2) Teacher behavior-student orientation positively impacts attitude toward learning English as a Foreign Language was accepted and confirmed with a standardized path coefficient of 0.194. The impact of English Teacher behavior student orientation on Attitude toward learning English as a Foreign Language is positively significant at the 0.001 level. H3) English self-concept positively and significantly impacts motivation for English was accepted and confirmed with a standardized path coefficient of 0.423. The impact of English self-concept on motivation for English is positively significant at the 0.001 level. H4) Teacher behavior student orientation has a positive and significant impact on English intentions was accepted and confirmed with a standardized path coefficient of 0.335. The impact of Teacher behavior student orientation on English intentions is positively significant at the 0.001 level. H5) English self-concept positively and significantly impacts English intentions was accepted and confirmed with a standardized path coefficient of 0.411. The impact of English self-concept on English intentions is positively significant at the 0.001 level.

H6) Teacher behavior student orientation has a positive and significant impact on motivation for English was accepted and confirmed with a standardized path coefficient of 0.347. The impact of Teacher behavior- student orientation on motivation for English is positively significant at the 0.001 level.

H7) Motivation for English has a positive and significant impact on attitude toward learning English as a foreign language was accepted with a standardized path coefficient of 0.271. The impact of motivation for English on attitude toward learning English as a foreign language is positively significant at the 0.001 level.

H8) English Intentions has a positive and significant impact on Attitude toward learning English as a Foreign Language was accepted with a standardized path coefficient of 0.279. The impact of English Intentions on attitude toward learning English as a foreign language is positively significant at the 0.001 level.

**Figure 3:**
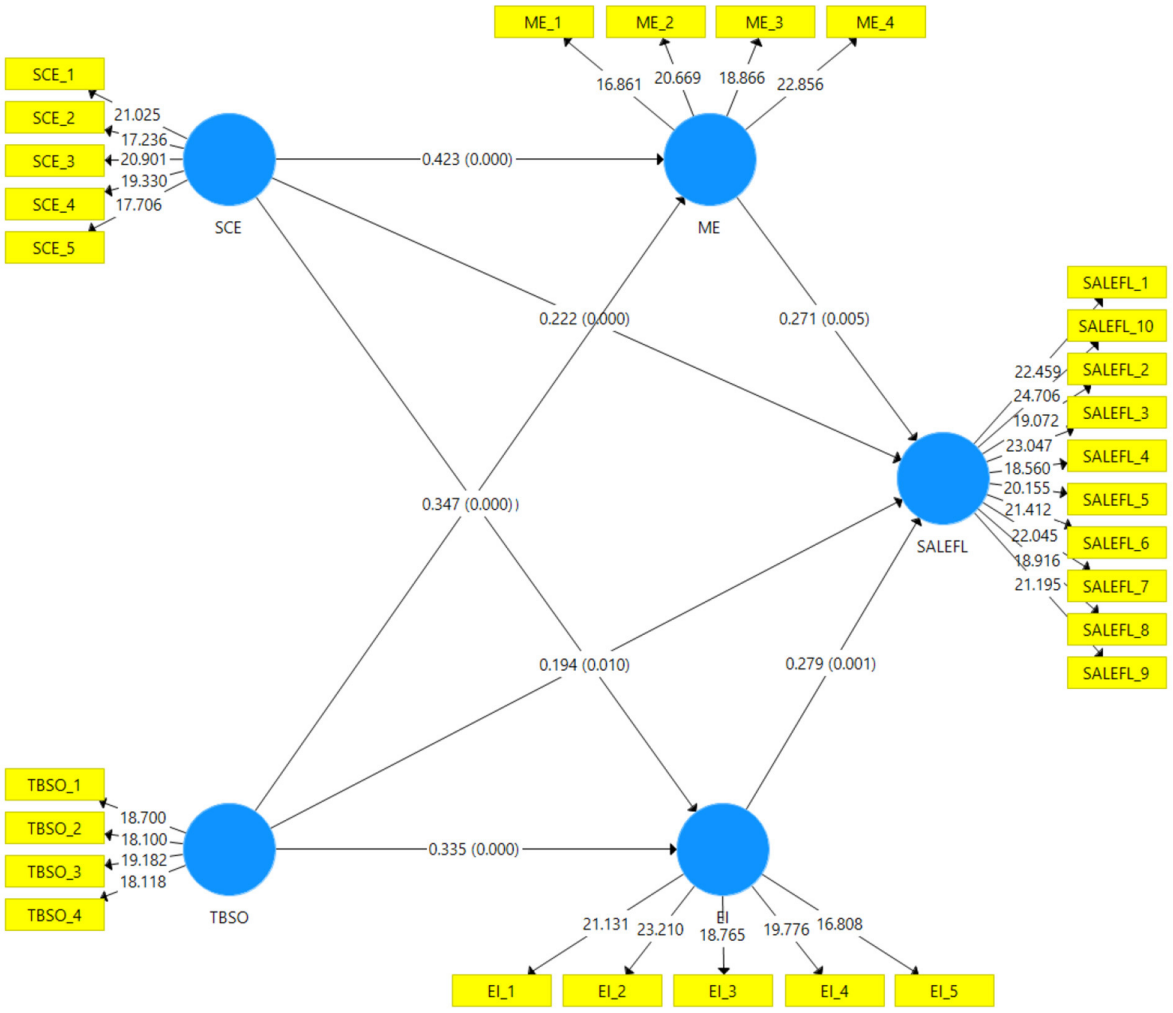
Structural model.

H7a) Motivation for English mediates the relationship between Self-concept English and attitude toward the learning of English as a Foreign Language was accepted at the beta value of 0.114, and the *p*-value was <0.05. H7b) Teacher behavior student orientation mediates the relationship between English Self-concept and attitude toward the learning of English as a foreign language was accepted at the beta value of 0.0.094, and the *p*-value was <0.05. Hypothesis H8(a) and H8(b) were accepted and confirmed at the beta value 0.115 and 0.093, respectively (as shown in Table 6). Figure 3 shows the study structural model outcomes.

**Table 6:** Hypotheses testing mediation effect.

**Hypothesis**	**Indirect**	**Std**.	**Std**.	**T**	**P**
	**Relationships**	***Beta***	**Error**	**Values**	**Values**
H7(a)	SCE → ME → SALEFL	0.114	0.047	2.424	*
H7(b)	TBSO → ME → SALEFL	0.094	0.041	2.299	*
H8(a)	SCE → EI → SALEFL	0.115	0.043	2.653	**
H8(b)	TBSO → EI → SALEFL	0.093	0.037	2.489	*

^*^Indicates significant paths: *p < 0.05, **p < 0.01. SCE, Self-concept English; ME, Motivation for English; SALEFL, Attitude toward the learning of English as a Foreign Language; TBSO, Teacher Behavior Student Orientation; EI, English Intentions.

Today, English has become a dominant language used worldwide. It is spoken and understood by the majority of people. It has made cross-culture education possible for the global communities (i.e., natives and non-natives). In particular, learning the English language has become a necessity in academics. Its increasing significance has made today's educators well aware of English teaching, concepts, and benefits. Indeed, in understanding its widespread, this study presents a generalized model illustrating the students' intention, motivation, and teachers' student orientation to influence the EFL process. Fundamentally, the study explores the factors that alter students' English language learning attitudes. Accordingly, section Conclusion provides a complete understanding of the benefit of EFL, thus significantly predicting students' intention to learn English as a foreign language. Altogether, this section highlights the study results compared to the previous works.

Undoubtedly, people have different reasons to learn the English language, but learning English as a foreign language demands a positive student attitude. Fundamentally, today's educators encourage the students to self-control their learning activity by regulating their skillsets and abilities. Therefore, for learning to be more effective, self-concept has drastically been adopted by the worldwide knowledge members (i.e., teachers and students). Education in all stages embraces the self-concept process as a beneficial instrument for enhancing students' English learning. Given the articulation, the study shows that self-concept allows the students to foster their EFL attitude and proficiency (68). Also, for directing the EFL attitude, teachers' self-belief and motivational behavior ensure the students' academic development. The teachers' motivational strategies enhance the students learning process. It encourages the student to indulge in self-learning, thus probing EFL behavior as an instrumental tool to achieve academic success (26). Indeed, it is no surprise that our results also support the previous literature, thereby accepting the assumptions made in H1 and H2.

Verily, learning English as a foreign language has become essential for students. English exposure is crucial for a learner's self-concept. In this regard, the study shows that self-concept makes students realize the value of learning the English language, thus motivating them to indulge in the English learning process (29). Also, the teachers teaching material, resources, and experiences help the students to gain proficiency in the English language. Language teachers are always aware of the career benefit that English proficiency offers to individuals. The teachers' student orientation behavior boosts the students' intention to learn English. In particular, by addressing the language concerns (e.g., teaching support), the study shows that students' learning motivation is the outcome of students' intention to learn (69). Also, the literature indicates that students' self-belief ensures effective English learning. In explaining this notion, the study shows that English self-concept increases the students' intention to learn English as a foreign language (17). However, surprisingly, the current study findings also support this literature, thus recording positive H3, H4, and H5.

Accordingly, positive teaching is a critical driving force that nurtures the classroom environment. Teachers play an instrumental role in elevating the students' motivation in the English teachings. Students' motivations in English as a foreign language influence students' learning process. Given the illustration, the teachers' support increases the students' autonomy and motivation for language learning in the future (70). Students' success is the output of individuals' motivation and effort. However, sometimes students don't realize the significance of learning the language. Therefore, they lack the motivation to further pursuing their studies. Given the articulation, the study shows that loss of motivation makes the students drop out when they experience difficulty in learning the language vocabulary (27). The poor learning motivation leads the individual to showcase a negative attitude, causing students failure in the course. In this regard, self-concept plays a vital role in boosting the students' motivation. The self-concept employed in language learning relates to the individual self-belief in increasing language proficiency. In explaining this notion, the study indicates a positive relationship between motivation, English self-concept, and students' English performance (71). Undoubtedly, our study results show positive outcomes, thus supporting H5 and H6.

Similarly, students' language learning intentions, attitudes, beliefs, and abilities foster the classroom environment. Hence, understanding this phenomenon makes the educationists predict the students' foreign language learning intentions. In the illustration, the study shows that students prefer to take foreign language learning classes over the other classes (72). Indeed, our study findings confirm the positive mediating role of student English learning intention (i.e., H7), as explained by previous studies. In conclusion, our research findings support all the hypotheses, thus recording positive results.

## Conclusion

In recent years, the global education sector has embraced the English language as a profound learning tool for increasing the students' motivation and intention. Apart from one's native language, English is the dominant learning of this modern world that has inevitably gained acceleration in the international world. English as a foreign language has brought different cultures together, thus encouraging the countries to mark it as a fundamental tool fostering the students' education. However, besides its increasing significance, English is a popular topic that has remained unaccepted in education.

Accordingly, the main objective of the present study was to investigate the factors stimulating the students' attitude toward learning English as a foreign language. These factors will lead the students to embrace English learning as an international language fostering self-concept and teachers' behavior student orientation. Moreover, the study also highlights the positive mediating effect of motivation and English intention against EFL (i.e., attitude toward learning English. Indeed, the results confirm that students' attitude toward the foreign learning language shows positive results for students, teachers, and education institutions. This study anticipates the findings to bring beneficial outcomes for the students, thus promoting EFL. Altogether, this incorporation appears to be a potential benefit in advancing the education sector. These insights develop a valuable contribution to the world's education sectors (i.e., eastern and western). In particular, the existing research gap showed that interest in the factors such as motivation and intention. Significantly, this study holds immense importance by empirically exploring the trajectories that influence students' perception of EFL learning.

## Data availability statement

The raw data supporting the conclusions of this article will be made available by the authors, without undue reservation.

## Ethics statement

Ethical review and approval was not required for the study on human participants in accordance with the local legislation and institutional requirements. The patients/participants provided their written informed consent to participate in this study.

## Author contributions

All authors listed have made a substantial, direct, and intellectual contribution to the work and approved it for publication.

## Conflict of interest

The authors declare that the research was conducted in the absence of any commercial or financial relationships that could be construed as a potential conflict of interest.

## Publisher's note

All claims expressed in this article are solely those of the authors and do not necessarily represent those of their affiliated organizations, or those of the publisher, the editors and the reviewers. Any product that may be evaluated in this article, or claim that may be made by its manufacturer, is not guaranteed or endorsed by the publisher.
